# Cyclic Stretch Force Induces Periodontal Ligament Cells to Secrete Exosomes That Suppress IL-1β Production Through the Inhibition of the NF-κB Signaling Pathway in Macrophages

**DOI:** 10.3389/fimmu.2019.01310

**Published:** 2019-06-20

**Authors:** Zhuyu Wang, Kentarou Maruyama, Yukihiko Sakisaka, Shigeki Suzuki, Hiroyuki Tada, Mizuki Suto, Masahiro Saito, Satoru Yamada, Eiji Nemoto

**Affiliations:** ^1^Department of Periodontology and Endodontology, Tohoku University Graduate School of Dentistry, Sendai, Japan; ^2^Department of Oral Immunology, Tohoku University Graduate School of Dentistry, Sendai, Japan; ^3^Department of Restorative Dentistry, Tohoku University Graduate School of Dentistry, Sendai, Japan

**Keywords:** exosomes, cyclic stretch, NF-κB signaling, inflammasome, periodontal ligament cells, macrophages

## Abstract

In the oral mechanical environment, periodontal ligament cells (PDL cells) contribute to maintaining periodontal tissue homeostasis. Recent studies showed that exosomes, which are small vesicles secreted by various types of cells, play a pivotal role in cell-to-cell communication in biological processes. We examined the secretion of exosomes from PDL cells stimulated with cyclic stretch and their role in the inflammatory response of macrophages using the human macrophage cell line THP-1 and human primary monocytes/macrophages. We prepared supernatants from human PDL cells (PDL-sup) stimulated with cyclic stretch. The treatment of macrophages with PDL-sup, but not PDL-sup from unstimulated PDL cells, inhibited the production of IL-1β in LPS/nigericin-stimulated macrophages. The pretreatment of PDL cells with GW4869, an inhibitor of exosome secretion, or siRNA for Rab27B, which controls exosome secretion, abrogated the inhibitory effects of PDL-sup. A transmission electron microscopy analysis demonstrated the existence of exosomes with diameters ranging between 30 and 100 nm in PDL-sup, suggesting that exosomes in PDL-sup contribute to this inhibition. An immunofluorescence microscopy analysis revealed that exosomes labeled with PKH67, a fluorescent dye, were incorporated by macrophages as early as 2 h after the addition of exosomes. Purified exosomes inhibited IL-1β production in LPS/nigericin-stimulated macrophages and the nuclear translocation of NF-κB as well as NF-κB p65 DNA-binding activity in LPS-stimulated macrophages, suggesting that exosomes suppress IL-1β production by inhibiting the NF-κB signaling pathway. Our results indicate that PDL cells in mechanical environments contribute to the maintenance of periodontal immune/inflammatory homeostasis by releasing exosomes.

## Introduction

Periodontal tissue is defined as tissue that supports teeth, and includes the root cementum, periodontal ligament (PDL), alveolar bone, and gingiva. The main functions of periodontal tissue are the attachment of tooth roots within the alveolar bone socket and the maintenance of mastication (1). The PDL is one of the main components of periodontal tissue and is a highly specialized, dense fibrous connective tissue between the cementum covering the root surface on the tooth and alveolar bone (1). It has many functions that include not only supporting teeth, but also contributing to the homeostasis of periodontal tissue and repair of damaged tissue. In a physiological environment, the PDL is exposed to mechanical loading derived from mastication, in which PDL cells respond to mechanical loading from the teeth, such as tension, compression, fluid shear, and hydrostatic force, and produce various components to regulate osteogenic-related signaling for the remodeling of periodontal tissue (2).

When infection or injury occurs, macrophages have an important role in protecting the host against infection and in tissue remodeling after inflammation (3). Monocytes originate from the bone marrow and circulate in the blood, undergoing differentiation into macrophages after infiltration of damaged/inflamed tissues (4). Macrophages have a central role in both the inflammatory process and repair of tissue damage. Among these processes, activation of inflammasomes occurs as part of the inflammatory response. Inflammasomes are cytosolic protein complexes that control activation of caspase-1, which is involved in activation of interleukin (IL)-1β and IL-18. Caspase-1 is also involved in an inflammatory mode of programmed cell death known as pyroptosis (5). Inflammasomes are formed by several members of the nucleotide-binding oligomerization domain (NOD)-like receptor (NLR) family as a response to diverse stimuli. Pyrin domain-containing 3 (NLRP3) is the best-characterized member of the NLR family, and it is involved in responses to various stimuli that include pathogen-associated molecules such as nigericin (6) and damage-associated molecular patterns such as extracellular adenosine triphosphate (ATP) (7). The NLRP3 inflammasome is comprised of NLRP3 protein itself, together with apoptotic speck-like protein (an adaptor protein with a caspase recruitment domain) and caspase-1. Activation of the NLRP3 inflammasome requires two steps. In the first step, induction of NLRP3 and pro-IL-1β expression occurs through transcriptional upregulation via the nuclear factor (NF)-κB signaling pathway (6). Subsequently, the components of the inflammasome are assembled in response to stimulation by microbial pathogens or endogenous danger signals, providing a platform for the activation of caspase-1, which cleaves pro-IL-1β and pro-IL-18 to produce the mature forms of these cytokines for secretion (5). However, sustained or excessive inflammasome activation in macrophages exacerbates pathological inflammation (8). Since periodontal lesions are often exposed to bacterial infection and/or injury, mechanisms that tightly regulate the NLRP3 inflammasomes of macrophages are required to maintain periodontal homeostasis. However, it currently remains unclear whether PDL cells are regulatory cells for immune/inflammatory homeostasis.

Exosomes, small membrane vesicles with diameters ranging between 30 and 100 nm, originate from multivesicular bodies, are secreted by a number of cell types, and exist in most body fluids. Exosomes are enriched in bioactive molecules, such as proteins, lipids, and nucleic acids, including mRNA, microRNA, and non-coding RNA, which are transferred between cells, thereby influencing the phenotypes and functions of target cells. There is increasing evidence to support exosomes released from different cell types functioning in cell-to-cell communication in physiological processes, such as cardiac remodeling (9, 10), airway remodeling (11, 12), bone remodeling (13–15), and tissue repair (16, 17), as well as pathological processes, including pro-inflammation (18–20)/anti-inflammation (21–23) and cancer (24). However, the involvement of exosomes in cell-to-cell communication in periodontal tissue has not yet been examined.

We hypothesized that exosomes play a pivotal role in the maintenance of immune/inflammatory homeostasis through intercellular communication between structural cells (e.g., PDL cells) and immune cells (e.g., macrophages) in periodontal tissue. In this study, we demonstrated that (1) cyclic stretch force strongly induced PDL cells to secrete exosomes, and that (2) exosomes inhibited NLRP3 inflammasome activation in LPS-stimulated human macrophages by attenuating the NF-κB signaling pathway.

## Materials and Methods

### Reagents

Nigericin, LPS (*Escherichia coli* O55:B5), fluorescein isothiocyanate (FITC)-conjugated LPS (*E. coli* O111:B4), cytochalasin D, phorbol-12-myristate-13-acetate (PMA), and dimethyl sulfoxide (DMSO) were obtained from Sigma (St. Louis, MO). GW4869 was purchased from Cayman Chemical (Ann Arbor, MI, USA). Recombinant human macrophage-colony stimulating factor (rhM-CSF) was purchased from Cell Signaling Technology (Danvers, MA, USA).

### Depletion of Exosomes From Fetal Bovine Serum (FBS) and Cell Culture Supernatants

Exosome-depleted FBS was prepared using the FBS Exosome Depletion Kit (Norgen, Thorold, ON, Canada) to remove exosomes originally contained in FBS. Briefly, 400 μl of ExoC buffer was added to 20 ml FBS mixed with 5 ml α-MEM medium. After an incubation at room temperature for 10 min, the mixture was transferred into the Maxi Spin column, then centrifuged at 500× *g* for 15 min to obtain the flowthrough, which contained exosome-depleted FBS. In the preparation of exosome-depleted cell culture supernatants, 30 μl of ExoC buffer was added to 2 ml of cell culture supernatants containing 10% (v/v) exosome-depleted FBS, and processing was then performed in a similar manner.

### Cell Lines and Culture

A mouse macrophage-like cell line (J774.1) was obtained from the Cell Resource Center for Biomedical Research, the Institute of Development, Aging, and Cancer, Tohoku University. The human monocyte-like cell line THP-1 was obtained from the American Type Culture Collection (Rockville, MD). These cell lines were cultured in RPMI 1640 medium (Gibco BRL, Rockville, MD) containing 10% heat-inactivated FBS (Gibco BRL) and antibiotics (100 U/ml penicillin G and 100 μg/ml streptomycin) under a humidified atmosphere (5% CO_2_). To induce the differentiation of THP-1 monocytes to macrophages, cells were incubated with 500 nM PMA for 4 h and cells that adhered to tissue culture plates were harvested by a treatment with 0.25% trypsin and 0.1% EDTA and then used in experiments. Only in the FITC-LPS binding assay, THP-1 monocytes were incubated with 500 nM PMA for 72 h to induce higher expression levels of CD14 because the binding activity of LPS to TLR4 is dependent on CD14 (25). Human primary monocytes from fresh peripheral blood were purchased from PromoCell GmbH (Heidelberg, Germany). Briefly, human CD14^+^ monocytes were isolated from fresh peripheral mononuclear cells using immunomagnetic particles specific for binding to CD14. To induce the differentiation of monocytes to macrophages, cells were incubated with 10 ng/ml rhM-CSF in RPMI1640 containing 10% FBS and antibiotics for 2 days. In the ELISA assay, differentiated THP-1 cells were seeded at 1.0 × 10^5^ or human primary monocytes/macrophages at 0.2 × 10^5^ on 96-well microplates. After a 24-h incubation in RPMI1640 with 10% FBS, cells were stimulated with appropriate stimulants for the indicated times.

### Primary Cells and Cell Culture

Human gingival fibroblasts (GF) were prepared from human gingival tissues obtained from clinically healthy patients (aged between 19 and 29 years old) at the time of third molar extraction without clinical signs of inflammation in periodontal tissues (26). Minced gingival tissues were cultured in α-MEM with 10% FBS and antibiotics until confluent cell monolayers had formed. Cells were used as confluent monolayers for experiments at subculture levels 3 through 10. Human PDL cells were prepared from the PDL of fully erupted lower third molar teeth (26). The PDL was dissected from the middle third of the root with a sharp blade. Tissue fragments were cultured in α-MEM with 10% FBS and antibodies until confluent cell monolayers had formed. Cells were used as confluent monolayers for experiments at subculture levels 3 through 10. Human dental pulp cells were obtained from the third molars of healthy individuals (aged between 19 and 29 years old) (27). Briefly, a groove was made in the buccal and occlusal tooth surfaces using a dental fissure bur and teeth were split using tooth forceps and a chisel. Dental pulp tissues were separated from teeth, cut into small pieces, and then cultured in α-MEM with 10% heat-inactivated FBS and penicillin/streptomycin, with a medium change every 3 days until confluent cell monolayers had formed. All of the above specimens were obtained at Tohoku University Hospital with informed consent. Experimental procedures were approved by the Ethical Review Board of Tohoku University Graduate School of Dentistry approval number 26–27.

### Cyclic Stretch

Cells were subjected to uniaxial cyclic stretch force by using the STB-140 STREX cell stretch system (STREX Co., Osaka, Japan). Silicon resin chambers (STB-CH-10.0, STREX Co.) were coated with 2 ml of 150 μg/ml type I atelocollagen (Atelo Cell®, KOKEN Co., Tokyo, Japan), followed by drying overnight on a clean bench. Human PDL fibroblasts, human GF, or human dental pulp fibroblasts (DPF) were seeded at 1.5 × 10^6^ cells in the collagen-coated chambers (area: 10.24 cm^2^) and incubated for 24 h in medium containing 10% FBS. Then the medium was exchanged for fresh medium containing 5% exosome-depleted FBS, and cells in the experimental groups were subjected to cyclic stretch (20% elongation at a frequency of 10 cycles/min for 24 h), while cells in the control groups were cultured for 24 h without cyclic stretch. All cells were maintained in a humidified atmosphere (5% CO_2_). After that period, the supernatant from the silicon resin chamber was prepared by centrifugation at 1,500 rpm at 4°C for 10 min to remove debris.

### Enzyme-Linked Immunosorbent Assay (ELISA) for Exosomes

The amounts of exosomes in supernatants were measured using the PS Capture™ Exosome ELISA Kit (Wako Pure Chemical Industries, Ltd., Osaka, Japan) according to the manufacturer's instructions. Briefly, supernatants were added to 96-well plates coated with a mouse anti-human CD63 antibody and incubated at room temperature for 1 h. After washing, the wells were incubated with horseradish peroxidase (HRP)-conjugated anti-mouse IgG Ab at room temperature for 1 h. The optical density at 450 nm was determined with the Softmax program (Molecular Devices).

### Isolation of Exosomes

Exosomes were isolated from cell culture supernatants using the PureExo® exosome isolation kit (101Bio, Mountain View, CA, USA) in accordance with the protocol. Briefly, cell culture supernatants were centrifuged at 3,000× g for 15 min to remove cellular debris. Solutions A, B, and C were added to a glass tube at a 1:1:4 ratio with a total volume of 0.75 ml and vortexed for 10 s. Two milliliters of the supernatant was added to this mixture and incubated at 4°C for 30 min, resulting in the formation of three-phase layers. After the top layer was discarded, the remainder was centrifuged at 5,000× *g* for 3 min, forming a new three-phase layer. The top and bottom layers were discarded and centrifugation was repeated. The middle fluffy layer was air-dried followed by re-suspension in 100 μL PBS with vigorous pipetting and a horizontal shaker. Then this suspension was subjected to centrifugation for 5 min at 5,000× *g*, after which the supernatant was processed on a PureExo® column and centrifuged at 1,000× *g* for 5 min. The flowthrough, which was the isolated pure exosome, was collected and stored at −80°C. The protein concentration of exosomes was measured using the DC Protein Assay® (Bio-Rad Laboratories, Hercules, CA, USA).

### Transmission Electron Microscopy (TEM)

Purified exosomes were inspected using HITACHI H-7600 at 100 kV (Hanaichi Ultrastructure Research Institute, Japan). Approximately 5 μl of a sample was placed on parafilm. A carbon-coated 400-mesh copper grid was then positioned on the top of the drop for 10 s and washed by a droplet of distilled water. The grid was contrasted by adding a drop of 2% uranyl acetate to the parafilm and incubating the grid on the top of the drop for 10 s. Excess liquid was gently removed using absorbing paper. After drying, it was submitted to TEM observations.

### Uptake of Exosomes by Macrophages

Purified exosomes were labeled with the PKH67 Green Fluorescent Cell Linker Kit® (Sigma-Aldrich, Oakville, ON, Canada) according to the manufacturer's protocol. Briefly, 2 μl of PKH67 dye was added into 250 μl Diluent C (Sigma-Aldrich). Exosomes in PBS were added into the PKH67 dye mixture at a volume of 3:1 and cultured at room temperature for 5 min. Unincorporated dye from labeled exosome preparations were removed by centrifugation using Exosome Spin Columns® (MW 3000; Invitrogen, Graiciuno, Lithuania). The labeled exosomes re-suspended in 20 μl of PBS, which corresponded to 1 ml of 1:2 diluted original culture supernatant, were incubated with THP-1 macrophages already seeded on a 35-mm poly-L-lysine-coated glass-bottomed dish (Matsunami Glass Ltd., Osaka, Japan) for 2 and 4 h, and staining was evaluated by immunofluorescence microscopy. Nuclei were stained with Hoechst 33342 (Immunochemistry Technologies, Bloomington, MN, USA) for 5 min.

### Gene Silencing With Small Interfering RNA (siRNA)

RNA sequences for targeting Rab27b by siRNA were selected using Enhanced siDirect, web-based target-specific siRNA design software. siRNAs were generated by RNAi Inc. (Tokyo, Japan). The control siRNA used in the present study was provided by RNAi Inc. Cells suspended in αMEM containing 5% exosome-depleted FBS were seeded on a collagen-coated silicon resin chamber with 10 nM siRNA against human *RAB27b* (sense strand 5′-GCAGUGGUGAGUUAAUCAUAG-3′ and antisense strand 5′-AUGAUUAACUCACCACUGCAC-3′) or control siRNA (sense strand 5′-GUACCGCACGUCAUUCGUAUC-3′ and antisense strand 5′-UACGAAUGACGUGCGGUACGU-3′) in the presence of Lipofectamine® RNAiMAX Transfection Reagent (Invitrogen, Carlsbad, CA, USA) for 24 h, according to the manufacturer's instructions. Cells were washed with PBS and cultured in αMEM containing 5% exosome-depleted FBS under a cyclic stretch stimulation for 24 h. The supernatant was then collected for subsequent experiments.

### Reverse Transcription and Real-Time Quantitative Polymerase Chain Reaction (RT-PCR)

For extraction of total RNA, Qiashredder and RNeasy® Kits (QIAGEN, Valencia, CA) were employed according to the directions of the manufacturers. The extracted RNA was treated with DNase (DNA-free™, Ambion Inc., Austin, TX) and was subjected to reverse transcription using a Transcriptor First Strand complementary DNA (cDNA) Synthesis Kit® (Roche Diagnostic Co., Indianapolis, IN) as per the manufacturer's instructions. Real-time PCR was done with cDNA converted from 50 ng of total RNA using amplification by 40 cycles of 95°C for 10 s, 55°C for 30 s, and 72°C for 30 s. The CFX96 Touch™ Real-Time PCR Detection System (Bio-Rad Laboratories, Hercules, CA) was used with iQ SYBR Green Supermix® (Bio-Rad Laboratories), an optimized volume of 3 mM MgCl_2_, and 500 nM of each primer. Expression of PCR products was normalized for that of glyceraldehyde 3-phosphate dehydrogenase (GAPDH). The sequences of the primers for genes encoding human IL-1β, IL-6, NLRP3, RAB27B, and GAPDH were as follows: *IL1B* (5′-TGTACCTGTCCTGCGTG-3′/5′-ACTGGGCAGACTCAAATTC-3′); *NLRP3* (5′-CGTGAGTCCCATTAAGATGGAGT-3′/5′-CCCGACAGTGGATATAGAACAGA-3′); *IL6* (5′-CTTTAAGGAGTTCCTGCAGTC-3′/5′-AATAGTGTCCTAACGCTCATAC-3′); *GAPDH* (5′-TGAACCATGAGAAGTATGACAACA-3′/5′-TCTTCTGGGTGGCAGTG-3′); *RAB27B* (5′-CACAAGGACCGAATGGATCT-3′/5′-CCATGGCGTCTCTGAAAAA-3′).

### Assay of IL-1β

After culture supernatants were harvested by centrifugation, IL-1β was measured using a human IL-1β Quantikine® ELISA kit (R&D Systems, Minneapolis, MN, USA) or mouse IL-1β ELISA Kit SimpleStep™ Kit (R&D Systems) according to the manufacturer's instructions. The amount of IL-1β was measured using the Softmax data analysis program (Molecular Devices, Menlo Park, CA).

### Western Blotting

Purified exosomes were initially treated with 2×Laemmli sample buffer (Bio-Rad Laboratories). After separation by sodium dodecyl sulfate-polyacrylamide gel electrophoresis, proteins were transferred to polyvinylidene difluoride membranes (ATTO, Tokyo, Japan) with a semidry transblot system (ATTO). Then the membranes were blocked for 1 h at room temperature with 0.5% (w/v) non-fat skim milk and 0.1% (v/v) Tween 20 in phosphate-buffered saline (PBS). Subsequently, membranes were incubated for 1 h at room temperature with a rabbit anti-exosome CD9 antibody (D8O1A, Cell Signaling Technology) diluted to 1:1,000, followed by incubation for 1 h at room temperature with HRP-conjugated goat anti-rabbit IgG antibody (Cell Signaling Technology) dilute to 1:2,000. After treatment with Western blotting detection reagent ECL Plus® (Amersham Pharmacia Biotech Inc., Piscataway, NJ), chemiluminescence was measured by using a ChemiDoc XRS Plus™ image analyzer (Bio-Rad Laboratories).

### Binding of LPS to Macrophages

Differentiated THP-1 macrophages were collected using cell dissociation solution (Sigma). Cells (5 × 10^5^ cells/0.1 ml) were incubated with RPMI1640 containing 5% FBS with 1 μg/ml of FITC-LPS in the presence or absence of 5 μg/ml of exosomes at 37°C for 15 min. After washing with cold PBS, cells were analyzed using a FACSCalibur® (Becton Dickinson, Mountain View, CA). Non-specific binding studies were performed with an excess (300 μg/ml) of unlabeled LPS (*E. coli* O55:B5). The binding of FITC-LPS was shown as a fluorescence histogram for each cell population.

### NF-κB p65 ELISA

After extraction of nuclear proteins, activated NF-κB was measured with a TransAM® NF-κB p65 transcription factor assay kit (Active Motif, Carlsbad, CA, USA) according to the manufacturer's instructions. Briefly, nuclear proteins were extracted using a Nuclear Extract Kit (Active Motif) according to the manufacturer's instructions. Nuclear extracts (10 μg/well) were added to 96-well plates coated with oligonucleotides containing the NF-κB consensus site (5′-GGGACTTTCC-3′) and incubated at room temperature for 1 h. After washing, the wells were incubated with NF-κB Ab at room temperature for 1 h followed by an incubation with HRP-conjugated anti-rabbit IgG at room temperature for 1 h. The optical density at 450 nm was determined with the Softmax data analysis program (Molecular Devices). The positive control was nuclear protein from Raji cells (5 μg/well) and the negative control contained no cell extract.

### Immunocytochemistry for NF-κB

After cells were fixed in ice-cold 100% methanol for 30 min at −20°C in a poly-L-lysine-coated glass-bottomed dish (Matsunami Glass Ltd.), permeabilization was done by incubation for 10 min in PBS containing 0.25% Triton X-100 (PBS-T). Then non-specific binding was blocked by incubation for 30 min in PBS-T containing 1% (w/v) bovine serum albumin (Sigma). Subsequently, incubation was done for 1 h with the primary rabbit anti-NF-κB p65 monoclonal antibody (D14E12, Cell Signaling Technology; 1:1,000 dilution), followed by incubation for 1 h with the secondary Alexa Fluor® 488 conjugated-goat anti-rabbit antibody (Invitrogen, Carlsbad, CA; 1:1,000 dilution). After nuclear staining by incubation for 1 min with 4′,6-diamidino-2-phenylindole (DAPI) (Invitrogen), evaluated by immunofluorescence microscopy was performed and cells showing nuclear translocation of NF-κB p65 were counted in three randomly selected fields containing ~100 cells each.

### Investigation of Pyroptosis

Staining with propidium iodide (PI) (Immunochemistry Technologies) was employed for detection of pyroptosis. In brief, cells were incubated for 5 min with 0.5% (v/v) PI, followed by incubation for 5 min with Hoechst 33342 to stain nuclei. Then immunofluorescence microscopy was performed and cells positive for PI staining were counted in three randomly selected fields containing ~100 cells each.

### Measurement of Lactate Dehydrogenase (LDH)

LDH activity was measured in culture supernatants by using a Cytotoxicity LDH Assay Kit-WST (Dojindo, Kumamoto, Japan) following the instructions of the manufacturer. LDH was measured in the culture supernatant of cells incubated with 10% (v/v) Lysis Buffer (Dojindo) as the positive control. The optical density at 490 nm (OD_490_) was determined with the Softmax program (Molecular Devices), and % release of LDH was calculated as follows: ([experimental OD_490_ – background OD_490_] /[positive control OD_490_ – background OD_490_]) × 100.

### Statistical Analysis

All experiments were repeated three times to test the reproducibility of the results, and representative results were shown. Results were shown as means ± SD. Differences between control and experimental groups were evaluated by the Kruskal-Wallis test with Steel's *post hoc* test. A *p*-value < 0.05 was regarded as indicating a significant difference.

## Results

### Cyclic Stretch Induces PDL Cells to Secrete Extracellular Inhibitory Factors for IL-1β Production by LPS-primed Macrophages

**Figure 1:**
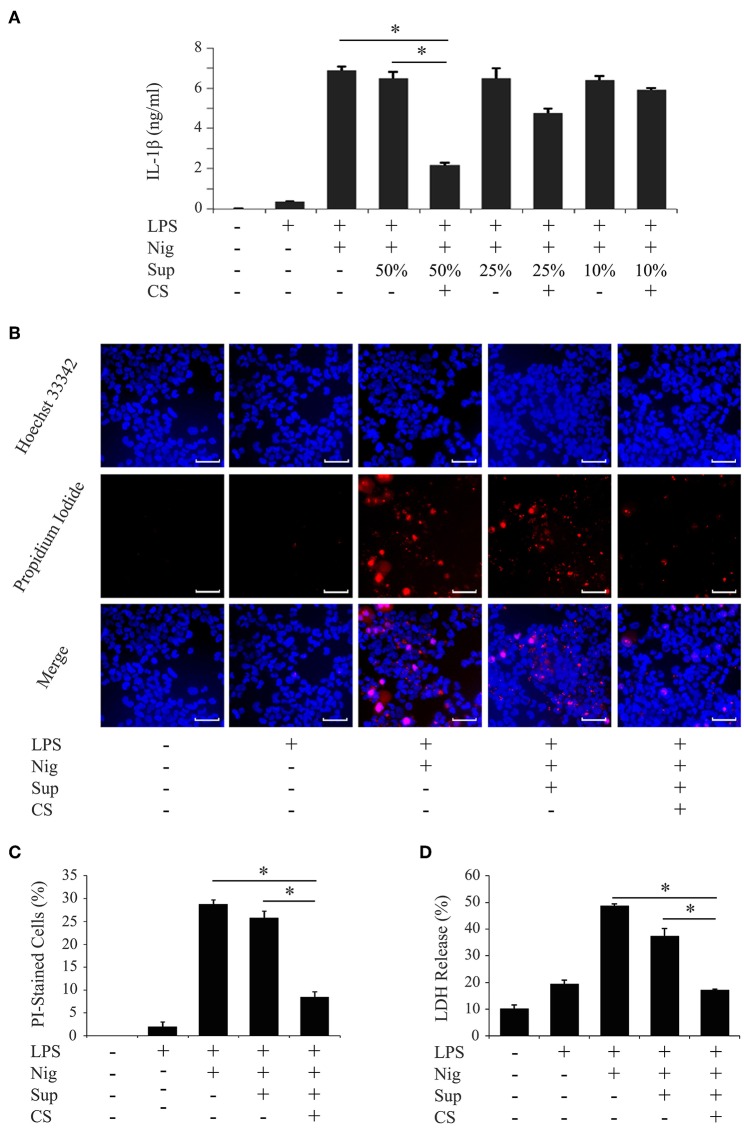
Cyclic stretch induces PDL cells to secrete extracellular inhibitory factors for IL-1β production in LPS-primed macrophages. PMA-pretreated THP-1 cells were primed with 1,000 ng/ml of *E. coli* LPS in the presence of the indicated % (v/v) of supernatants (Sup) from cyclic stretch (CS)-exposed PDL cells for 4 h followed by a stimulation with 10 μM nigericin (Nig) for 2 h in the continuous presence of LPS and Sup. **(A)** The amount of IL-1β in Sup from THP-1 macrophages was measured by ELISA. **(B)** THP-1 macrophages were labeled with PI (red: shown in the middle panel) and nuclei were visualized by staining with Hoechst 33342 (blue: shown in the upper panel). Merged images were shown in the lower panel. (Magnification: ×100, scale bars are 50 μm). **(C)** THP-1 macrophages stained by PI in three randomly selected fields (each containing ~100 cells) were quantified. **(D)** LDH activity in Sup from THP-1 macrophages was measured using a LDH assay kit. Representative data from three separate experiments were shown as the means ± SD of triplicate assays and significance was indicated (*p < 0.05 significantly different from the control).

We investigated whether cyclic stretch acted on human PDL cells to promote regulation of the inflammatory response of macrophages using the THP-1 human monocyte-like cell line. PMA-pretreated THP-1 cells were primed by incubation for 4 h with *E. coli* LPS (1,000 ng/ml), followed by stimulation for 2 h with the NLRP3 inflammasome activator nigericin (10 μM) plus LPS. As shown in Figure 1A, a significant amount of IL-1β (6890.71 ± 215.18 pg/ml) was detected. Supernatants from PDL cells, which were exposed to cyclic stretch for 24 h, were then added to the macrophage culture together with LPS, and IL-1β production was evaluated. Figure 1A shows that the addition of the supernatant at a concentration of 50% (v/v) markedly decreased IL-1β production, whereas the supernatant from the normal culture of PDL cells did not. This inhibitory effect occurred in a dose-dependent manner and was undetected at a concentration of 10% (v/v). Thus, the concentration of 50% (v/v) supernatant was applied in subsequent experiments. The inhibitory effects of the supernatants of PDL cells were reproduced in three different donors (Table 1, upper three rows). Pyroptosis is a mode of programmed cell death mediated by caspase-1, in which rupture of the plasma membrane follows inflammasome activation (28). We examined whether culture supernatants of PDL cells exposed to cyclic stretch inhibited the induction of pyroptosis by nigericin in macrophages primed with LPS by detecting dead cells stained by PI. Figures 1B,C show that exposure to LPS and nigericin increased the number of PI-stained cells, while exposure to culture supernatant reduced the number of PI-stained cells. Figure 1D shows that release of LDH by macrophages was greater after stimulation with LPS and nigericin compared to unstimulated control cells or after exposure to LPS alone (48.71 ± 0.73 vs. 19.44 ± 1.43% and 10.14 ± 1.51%, respectively), while supernatants from PDL cells exposed to cyclic stretch significantly inhibited the release of LDH, suggesting the suppression of nigericin-induced pyroptosis in LPS-primed macrophages. These results indicate that cyclic stretch induces PDL cells to secrete extracellular inhibitory factors for inflammasome activation in macrophages. Similar results were obtained using the mouse macrophage-like cell line J774.1; the addition of the supernatant at a concentration of 50% (v/v) markedly decreased IL-1β production (Supplementary Figure 1A), the number of PI-staining cells (Supplementary Figure 1B), and the release of LDH (Supplementary Figure 1C) in LPS-primed J774.1 macrophages. Among the cells related to teeth/periodontal tissues, only PDL cells, not GF or DPF, are physiologically exposed to cyclic stretch from daily mastication. Therefore, we investigated whether cyclic stretch exerts similar effects on other tooth/periodontal tissue-related cells. We prepared supernatants from cyclic stretch-exposed GF and DPF from two to three different donors and examined IL-1β production in the same manner as that described for PDL cells. Table 1 shows that supernatants from cyclic stretch-exposed GF and DPF had no significant effect on IL-1β production in LPS-primed macrophages from the respective normally cultured fibroblasts. These results suggest that the inhibitory effects of cyclic stretch are not universal in tooth/periodontal tissue-related cells and may be selective for PDL and cells.

**Table 1:** Effects of supernatants from tooth/periodontal tissue-related cells on IL-1β production in macrophages.

		**IL-1β** **(pg/ml)**		
		**LPS** **(+), Nig** **(+)**		
		**Fresh medium**	**Supernatant**	
**Donor**	**Cell type**		**Cyclic stretch (–)**	**Cyclic stretch **(+)****
1	PDL	6890.71 ± 215.18	6665.10 ± 309.87	2194.46 ± 123.73^*^^§^
2	PDL	7050.14 ± 294.60	7273.18 ± 134.84	2500.60 ± 195.94^*^^§^
3	PDL	9025.45 ± 788.72	8791.55 ± 467.35	1944.11 ± 120.88^*^^§^
4	GF	9356.18 ± 83.95	9665.98 ± 480.06	8755.10 ± 119.37^ns^
5	GF	8798.47 ± 160.72	8603.36 ± 594.68	8372.77 ± 415.27^ns^
6	GF	9517.85 ± 122.94	9759.40 ± 29.25	8825.69 ± 375.06^ns^
7	DPF	12261.33 ± 318.53	12029.12 ± 505.58	11219.09 ± 576.23^ns^
8	DPF	11196.40 ± 202.42	11611.57 ± 841.34	11052.36 ± 87.84^ns^

PMA-pretreated THP-1 cells were primed with 1,000 ng/ml of E. coli LPS in the presence of 50% (v/v) fresh medium (as positive control) or 50% (v/v) supernatants from cyclic stretch-exposed PDL cells (three different donors), gingival fibroblasts (three different donors), or dental pulp fibroblasts (two different donors) for 4 h, followed by a stimulation with 10 μM nigericin for 2 h in the continuous presence of LPS and the respective supernatants. The amount of IL-1β in supernatants from THP-1 macrophages was measured by ELISA. Representative data from three separate experiments were shown as the means ± SD of triplicate assays and significance was indicated (

*: *p < 0.05 significantly different from cyclic stretch (–) supernatant-treated cells*,

§: *p < 0.05 significantly different from fresh medium-treated cells, ns: p > 0.05, not significantly different from cyclic stretch (–) supernatant-treated or fresh medium-treated cells)*.

### Cyclic Stretch Induces PDL Cells to Secrete Exosomes

**Figure 2:**
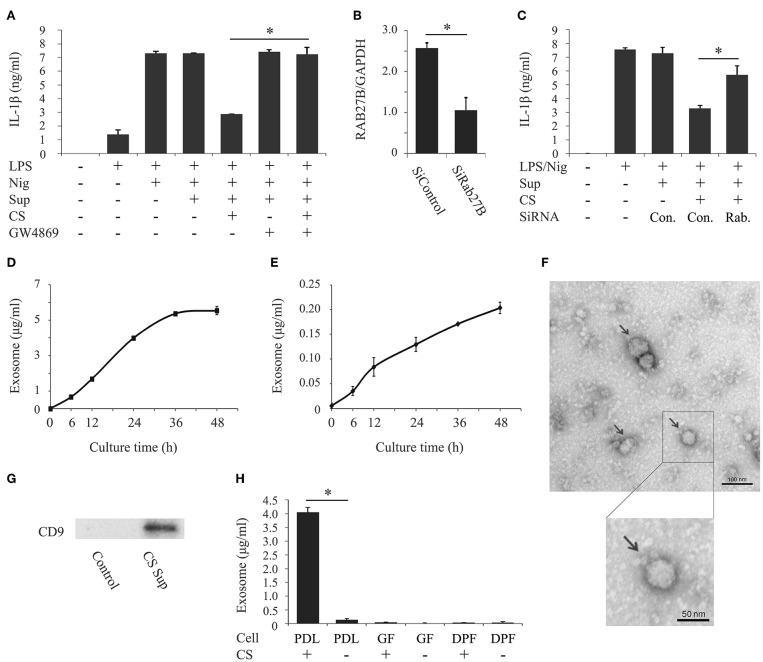
Cyclic stretch induces PDL cells to secrete exosomes. **(A)** Supernatants (Sup) were prepared from PDL cells exposed to cyclic stretch (CS) in the presence of 10 μM GW4869 for 24 h. All Sup, including the control, were adjusted to contain 0.69% (v/v) DMSO in culture medium during the PDL cell culture. PMA-pretreated THP-1 cells were primed with 1,000 ng/ml of *E. coli* LPS in the presence of 50% (v/v) of the Sup for 4 h followed by a stimulation with 10 μM nigericin (Nig) for 2 h in the continuous presence of LPS and Sup. The amount of IL-1β in Sup from THP-1 macrophages was measured by ELISA. **(B)** Sub-confluent cells were transfected with siRNA against human RAB27B (SiRAB27B) or negative control siRNA (SiControl) and cultured for 24 h. Total cellular RNA was extracted and transcripts of *RAB27B* were analyzed by real-time PCR. **(C)** Following transfection, Sup were prepared from PDL cells exposed to CS for 24 h. THP-1 macrophages were primed with 1,000 ng/ml of *E. coli* LPS in the presence of 50% (v/v) of the Sup for 4 h followed by a stimulation with 10 μM Nig for 2 h in the continuous presence of LPS and Sup. The amount of IL-1β in Sup from THP-1 macrophages was measured by ELISA. **(D,E)** Sup were prepared from PDL cells with/without CS for the indicated times. The amounts of exosomes in the Sup were measured using an exosome ELISA kit. **(F)** Exosomes purified from the Sup of CS-exposed PDL cells were observed by TEM (Magnification: ×10,000 and ×15,000, scale bars are 100 and 50 nm). Arrows indicate exosomes with diameters of ~30–100 nm. **(G)** Exosomes purified from Sup from CS-exposed PDL cells were analyzed by Western blotting with anti-CD9 mAb (molecular mass: 25 kDa). The exosome fraction from PDL cells without CS was used as a control. **(H)** Sup were prepared from PDL cells, gingival fibroblasts (GF), or dental pulp fibroblasts (DPF) exposed to CS for 24 h. The amounts of exosomes in the Sup were measured using an exosome ELISA kit. Representative data from three separate experiments were shown as the means ± SD of triplicate assays and significance is indicated (*P < 0.05 significantly different from the control).

Previous studies reported that PDL cells release various bioactive molecules when stimulated with cyclic stretch, such as proteins, peptides, nucleotides, and prostaglandins (29–31). Exosomes have recently been implicated as an important communication tool among various cell types. Although it currently remains unclear whether cyclic stretch-exposed PDL cells secrete exosomes, we investigated if exosomes contribute to the inhibition of IL-1β production. We prepared the supernatant from PDL cells exposed to cyclic stretch in the presence of GW4869, a chemical neutral sphingomyelinase-2 inhibitor, which inhibits exosome secretion (32), and tested the effects of the supernatant on IL-1β production in LPS-primed macrophages. Figure 2A shows that the treatment with GW4869 restored the inhibitory effects of the supernatant on IL-1β production. Note that the GW4869 treatment in the normal culture of PDL cells had no effects on IL-1β production. Furthermore, siRNA was conducted on PDL cells to silence Rab27B, which controls exosome secretion (33). Figure 2B shows that the expression of Rab27B mRNA in cells pretreated with siRab27B for 24 h was significantly weaker (64% reduction) than that in control-siRNA-pretreated PDL cells. Figure 2C shows that the silencing of Rab27B significantly recovered the inhibitory effects of the supernatant on IL-1β production. These results suggest that exosomes, which may be secreted from cyclic stretch-exposed PDL cells, contribute to inhibitory effects on IL-1β production in LPS-primed macrophages. We then investigated whether exosomes are detectable in the supernatants of cyclic stretch-exposed PDL cells using ELISA, the plates of which were coated with the antibody for CD63 as one of the exosome markers. Figures 2D, E show that significant amounts of exosomes were detected in supernatants from cyclic stretch-exposed PDL cells in a time-dependent manner with a plateau of 36 h, whereas only a marginal amount of exosomes was detected from the normal culture of PDL cells (~4,000 ng/ml for cyclic stretch-exposed PDL cells vs. 120 ng/ml in a normal culture of PDL cells at 24 h). Exosomes purified from supernatants were verified by TEM. The TEM analysis showed that the diameters of exosomes ranged between 30 and 100 nm with a median average of 50 nm, which is consistent with the characteristic size range of exosomes (Figure 2F). The expression of CD9, an exosomal marker, was detected on exosomes purified from supernatants using a Western blot analysis (Figure 2G). As expected from the results shown in Table 1, exosomes were not detected in the supernatants of GF or DPF regardless of whether cyclic stretch was applied (Figure 2H).

### The Uptake of Exosomes by Macrophages

**Figure 3:**
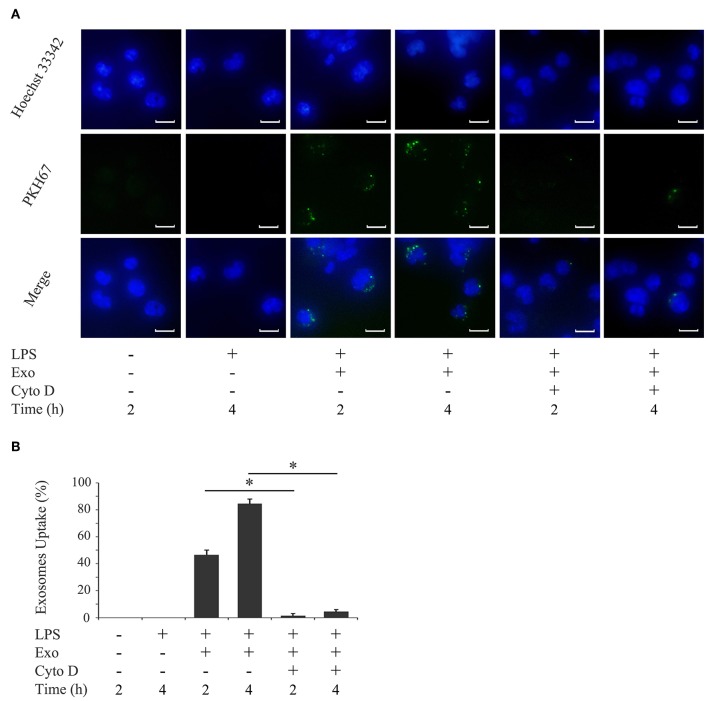
Exosomes are taken up by macrophages. THP-1 macrophages were incubated with 1,000 ng/ml of *E. coli* LPS in the presence of PKH67-labeled exosomes (Exo) with/without 10 μM cytochalasin D (Cyto D) for the indicated times. **(A)** Exo taken up by THP-1 macrophages (light green: in the middle panel) were detected by immunostaining after 2 and 4 h. Nuclei were visualized by staining with Hoechst 33342 (blue: shown in the upper panel). Merged images were shown in the lower panel (magnification: ×400, scale bars are 10 μm). **(B)** Cells stained with PKH67-labeled Exo in three randomly selected fields (each containing ~100 cells) were quantified. Representative data from three separate experiments were shown as the means ± SD of triplicate assays and significance is indicated (*P < 0.05 significantly different from the control).

To examine the uptake of purified exosomes by macrophages, exosomes were labeled with PKH67, a fluorescent dye that is incorporated into the lipid membrane of exosome vesicles. An immunofluorescence microscopy analysis revealed that the uptake of exosomes by THP-1 macrophages occurred as early as 2 h after the addition of exosomes and increased by 4 h (Figures 3A,B). The uptake of exosomes was not affected by the activation status of THP-1 macrophages (Supplementary Figures 2A,B). Since actin polymerization is required for the uptake of exosomes by macrophages (34), THP-1 macrophages were pretreated with cytochalasin D, an inhibitor of actin polymerization. Figures 3A,B (the right two columns) show that exosome uptake was mostly inhibited over time. The inhibition of exosome entry was not due to toxic effects of cytochalasin D on macrophages, as cytochalasin D did not affect macrophage viability (data not shown).

### Exosomes From Cyclic Stretch-Exposed PDL Cells Inhibit IL-1β Production and Pyroptosis in LPS-primed Macrophages

**Figure 4:**
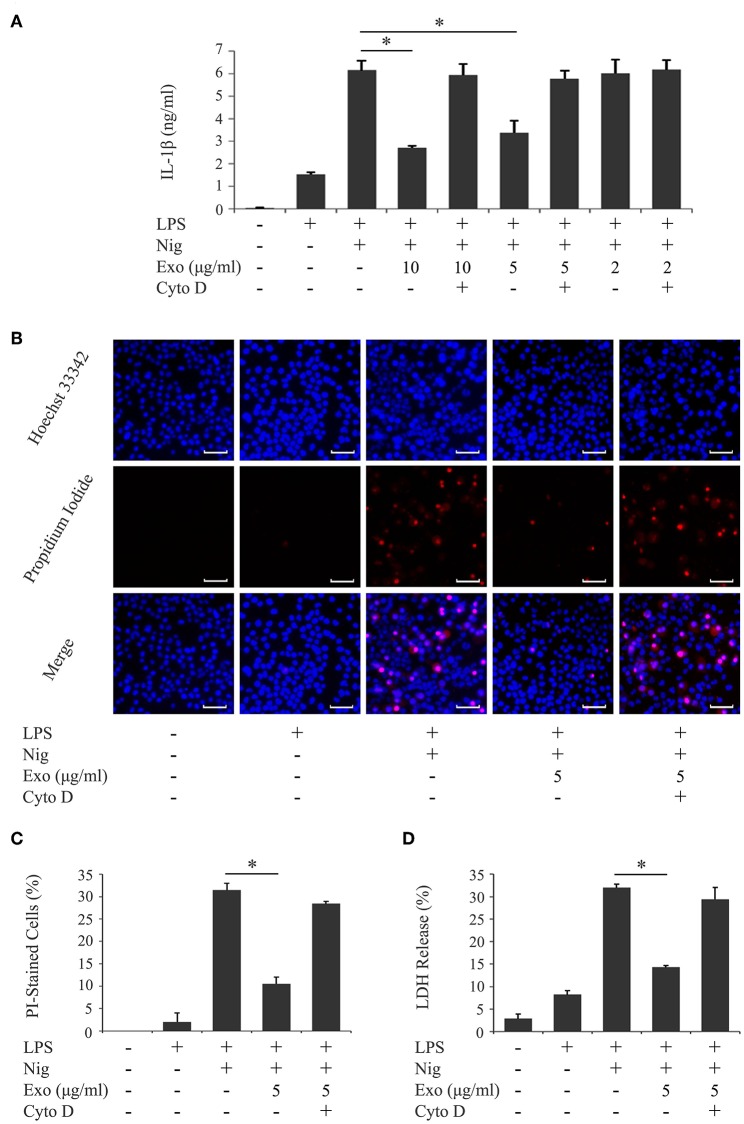
Exosomes from cyclic stretch-exposed PDL cells inhibit IL-1β production and pyroptosis in LPS-primed THP-1 macrophages. THP-1 macrophages were primed with 1,000 ng/ml of *E. coli* LPS in the presence of the indicated concentration of exosomes (Exo) with/without 10 μM cytochalasin D (Cyto D) for 4 h, followed by a stimulation with 10 μM nigericin (Nig) for 2 h in the continuous presence of Exo and LPS. **(A)** The amount of IL-1β in supernatants from THP-1 macrophages was measured by ELISA. **(B)** Cells were labeled with PI (red: shown in the middle panel) and nuclei were visualized by staining with Hoechst 33342 (blue: shown in the upper panel). Merged images were shown in the lower panel. (Magnification: ×100). **(C)** Cells stained by PI in three randomly selected fields (each containing ~100 cells) were quantified. **(D)** LDH activity in supernatants from THP-1 macrophages was measured using a LDH assay kit. Representative data of three separate experiments were shown as the means ± SD of triplicate assays and significance was indicated (*p < 0.05 significantly different from the control).

**Figure 5:**
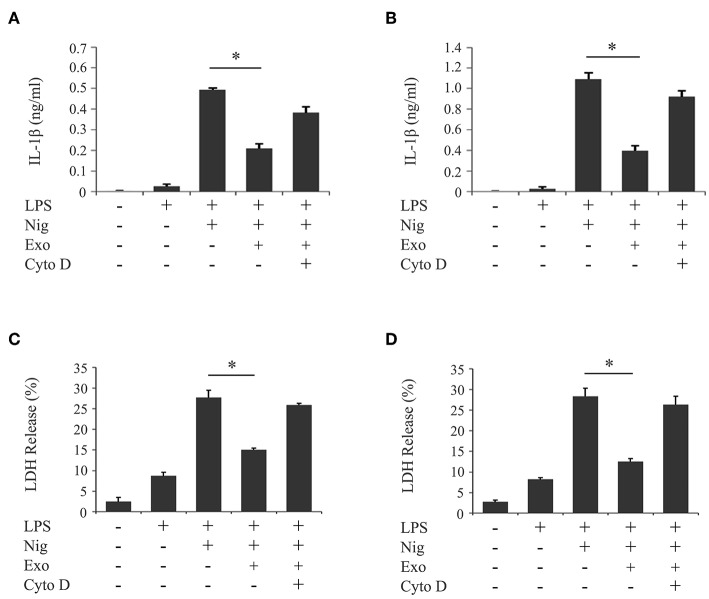
Exosomes from cyclic stretch-exposed PDL cells inhibit IL-1β production and pyroptosis in LPS-primed human primary monocytes/macrophages. Human primary monocytes were incubated with or without 10 ng/ml rhM-CSF for 2 days (referred to as human primary macrophages or human primary monocytes, respectively). These cells were primed with 1,000 ng/ml of *E. coli* LPS in the presence of 5 μg/ml exosomes (Exo) with/without 10 μM cytochalasin D (Cyto D) for 4 h, followed by a stimulation with 10 μM nigericin (Nig) for 2 h in the continuous presence of Exo and LPS. The amount of IL-1β in supernatants from human primary monocytes **(A)**/macrophages **(B)** was measured by ELISA. LDH activity in supernatants from human primary monocytes **(C)**/macrophages **(D)** was measured using a LDH assay kit. Representative data of three separate experiments were shown as the means ± SD of triplicate assays and significance was indicated (*p < 0.05 significantly different from the control).

We investigated the influence of purified exosomes on production of IL-1β by LPS-primed macrophages. Purified exosomes were added to the THP-1 macrophage culture together with LPS, and IL-1β production was evaluated in the same manner as in Figure 1A. Figure 4A shows that exosomes at a concentration of 5.0 μg/ml, which corresponds to the exosome content in the original supernatant from cyclic stretch-exposed PDL cells, markedly inhibited IL-1β production. This inhibitory effect of exosomes occurred in a dose-dependent manner and was restored by the pretreatment of macrophages with cytochalasin D. A similar result was obtained using the mouse macrophage-like cell line J774.1 (Supplementary Figure 3). Furthermore, the addition of purified exosomes to LPS/nigericin-stimulated THP-1 macrophages reduced the number of PI-stained cells (Figures 4B,C) as well as LDH release (Figure 4D), suggesting that exosomes inhibited pyroptosis of LPS-primed macrophages induced by nigericin. The inhibitory effects of exosomes were restored by the pretreatment of macrophages with cytochalasin D. These results suggest that exosomes from cyclic stretch-exposed PDL cells inhibit NLRP3-inflammasome activation in macrophages. Although the THP-1 cell line is a valuable tool for investigating human monocyte/macrophage functions under healthy and disease conditions, it is limited as a model for human primary monocytes and macrophages (35, 36). We investigated whether the inhibitory effects of exosomes were reproduced in human primary monocytes and macrophages in the same manner as in Figure 4, and obtained similar results showing that exosomes inhibited IL-1β production and LDH release in each cell, as shown in Figure 5. The amount of IL-1β produced from human primary macrophages was similar to that from PMA-stimulated THP-1 macrophages in terms of per cell number.

### Exosomes From Cyclic Stretch-exposed PDL Cells Inhibit the LPS-induced NF-κB Signaling Pathway in LPS-primed Macrophages

**Figure 6:**
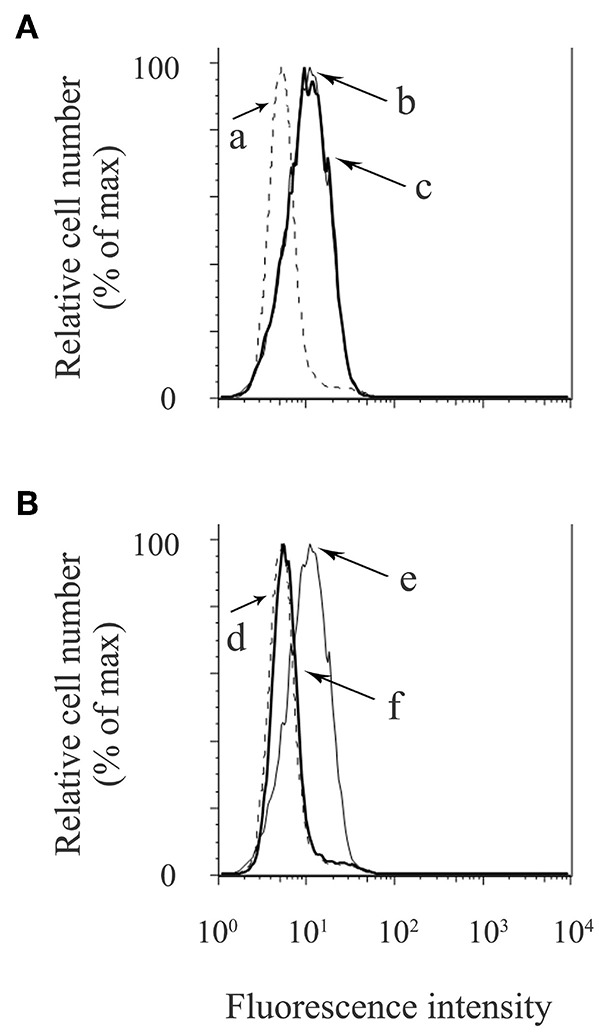
Exosomes from cyclic stretch-exposed PDL cells do not affect the binding activity of LPS to macrophages. THP-1 macrophages were incubated with 1 μg/ml of FITC-LPS in the presence or absence of 5 μg/ml of exosomes at 37°C for 15 min. Cells were analyzed using a FACSCalibur cytometer®. **(A,B)** Fluorescence histograms are shown for each cell population. a, No FITC-LPS (dash line); b, FITC-LPS without exosomes (solid line); c, FITC-LPS with exosomes (bold solid line); d, No FITC-LPS (dash line); e, FITC-LPS (solid line); f, FITC-LPS with an excess of non-labeled LPS (bold solid line). Representative data of three separate experiments are shown.

**Figure 7:**
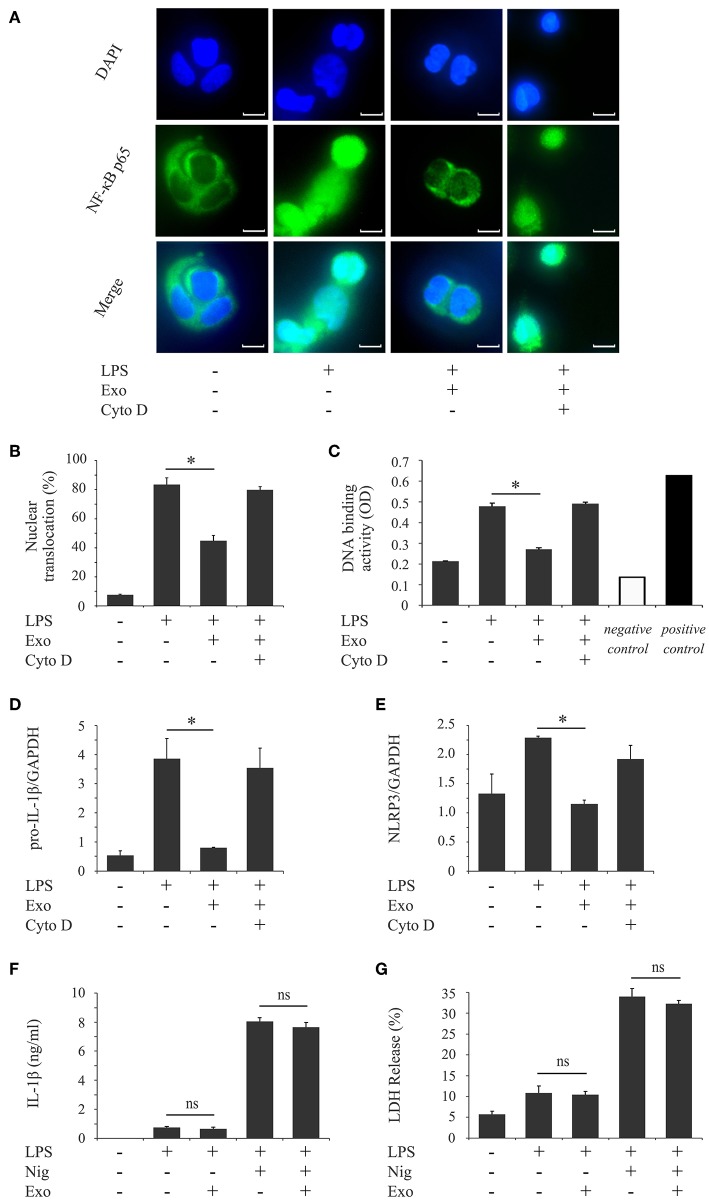
Exosomes from cyclic stretch-exposed PDL cells inhibit the LPS-induced NF-κB signaling pathway in LPS-primed macrophages. **(A–E)** THP-1 macrophages were primed with 1,000 ng/ml of *E. coli* LPS in the presence of 5 μg/ml of exosomes (Exo) with/without 10 μM cytochalasin D (Cyto D) for 4 h. **(A)** The nuclear translocation of NF-κB p65 (green: shown in the middle panel) was detected by immunostaining. Nuclei were visualized by staining with DAPI (blue: shown in the upper panel). Merged images were shown in the lower panel (magnification: ×400; scale bars are 10 μm). **(B)** Cells exhibiting the nuclear translocation of NF-κB p65 in three randomly selected fields (each containing ~100 cells) were quantified. **(C)** Nuclear proteins were extracted from cells and a NF-κB ELISA assay was performed. The positive control was provided by 5 μg of the nuclear extract of Raji cells. A sample with no cell extract was used as a negative control. **(D,E)** Total cellular RNA was extracted, and transcripts of *PRO-IL-1*β **(D)** and *NLRP3* **(E)** were analyzed by real-time quantitative PCR. **(F,G)** THP-1 macrophages were primed with 1,000 ng/ml of *E. coli* LPS for 4 h followed by a treatment with/without 5 μg/ml Exo for 1 h, and then stimulated with/without 10 μM nigericin for an additional 2 h. **(F)** The amount of IL-1β in supernatants from THP-1 macrophages was measured by ELISA. **(G)** LDH activity in supernatants from THP-1 macrophages was measured using a LDH assay kit. Representative data from three separate experiments were shown as the means ± SD of triplicate assays and significance is indicated (*P < 0.05 significantly different from the control; ns, not significant).

NLRP3-inflammasome activation involves two phases: LPS priming and nigericin triggering (5). LPS initially binds to the cell surface receptor, toll-like receptor 4 (TLR4). When macrophages are primed by LPS, activation of NF-κB signaling promotes expression of inflammasome-related molecules, including pro-IL-1β and NLRP3 (37). The NF-κB dimer p50/p65 usually exists in the cytoplasm where it is bound to an inhibitory protein (IκB). LPS stimulates the degradation of IκB, thus freeing NF-κB to undergo nuclear translocation and binding to specific response sequences in the promoter regions of its downstream target genes (38). We examined whether exosomes affect the binding activity of LPS to the macrophage cell surface. Figure 6A shows that FITC-LPS significantly bound to differentiated THP-1 macrophages (histogram: b) and that the addition of exosomes did not alter the binding of FITC-LPS to macrophages (histogram: c). This binding was specific for LPS because the binding of FITC-LPS (histogram: e) was completely abolished by the addition of an excess of non-labeled LPS (histogram: f), as shown in Figure 6B. We then investigated whether exosomes regulate the NF-κB signaling pathway during LPS priming. In Figures 7A,B, immunohistochemistry demonstrates that purified exosomes inhibited the translocation of NF-κB in response to LPS stimulation. The inhibitory effect of exosomes was restored by pretreatment of macrophages with cytochalasin D. We also performed ELISA of nuclear extracts to assess the binding of NF-κB p65 to specific DNA sequences. As shown in Figure 7C, exosomes significantly inhibited LPS-induced binding of NF-κB, which was restored by pretreatment of macrophages with cytochalasin D. These results suggest that purified exosomes from PDL cells inhibit the NF-κB signaling pathway during the LPS priming of macrophages. Similar results in terms of the nuclear translocation of NF-κB as well as NF-κB p65 DNA-binding activity were obtained using J774.1 macrophages (Supplementary Figures 4A–C). This is supported by our results (Figures 7D,E) showing that LPS-induced expression of NLRP3 and pro-IL-1β, which is dependent on NF-κB signaling (6), was inhibited by exosomes, but was restored by pretreatment of macrophages with cytochalasin D. We then investigated whether exosomes regulate the nigericin-triggering phase of NLRP3-inflammasome activation. Purified exosomes were added to THP-1 macrophages after LPS priming and then stimulated with nigericin. Figures 7F,G (the first and second bars from the right side) show that exosomes did not affect nigericin-induced IL-1β production or LDH release from LPS-primed macrophages. On the other hand, a previous study reported that exosomes belong to the category of damage-associated molecular patterns (DAMPs) (39), suggesting that exosomes act as potential independent signal 2. We examined this possibility by adding exosomes instead of nigericin after priming THP-1 macrophages with LPS. Figures 7F,G (the second and third bars from the left side) show that exosomes did not have any effect on IL-1β production or LDH release in THP-1 macrophages, suggesting that exosomes do not have roles as DAMPs. These results indicate that exosomes inhibit IL-1β production and pyroptosis in macrophages by suppressing the NF-κB signaling pathway in the LPS-priming phase, but independently of the nigericin-triggering phase.

## Discussion

**Figure 8:**
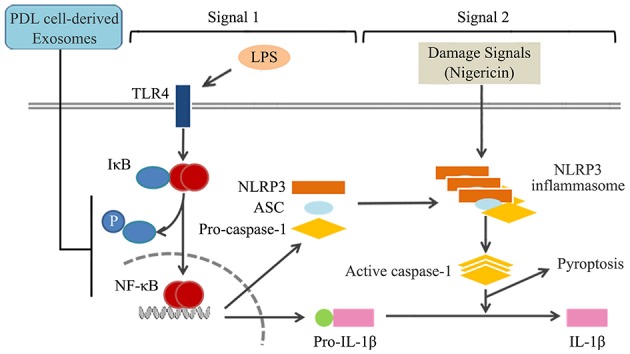
Summary of mechanisms by which exosomes from cyclic stretch-exposed PDL cells negatively regulate IL-1β production in macrophages. The treatment of macrophages with LPS activates NF-κB signaling via TLR4 (Signal 1), and induces the expression of NLRP3 and pro-IL-1β. NLRP3 inflammasome components, which consist of NLRP3, apoptotic speck-like protein (ASC), and caspase-1, are assembled after being exposed to nigericin (Signal 2), and induce the activation of caspase-1, which leads to the production of IL-1β and pyroptosis. Exosomes from cyclic stretch-exposed PDL cells inhibit the activation of NF-κB signaling without affecting the binding activity of LPS to macrophages (Signal 1), but do not interfere with Signal 2, which is followed by the negative regulation of IL-1β production and pyroptosis.

We demonstrated that cyclic stretch, which mimics the physiological mechanical environment of the PDL, induces PDL cells to secrete exosomes. We also demonstrated that exosomes inhibited NLRP3 inflammasome signaling in human macrophages primed with LPS by inhibiting the NF-κB signaling pathway without affecting the binding of LPS to macrophages, as shown in Figure 8.

The PDL connects the roots of the teeth to the alveolar bone and is affected by mechanical force during mastication. PDL cells function as professional mechano-sensing cells that respond to loading on the teeth and convert mechanical signals to chemical signals involved in regulation of various genetic and biochemical pathways (40). It has generally been accepted that mechanical force induces PDL cells to secrete a wide range of growth factors, which regulate the physiological remodeling of periodontal tissue, thereby maintaining the balance between periodontal hard tissues (29, 41–43) and soft tissues (30, 31). However, few reports are currently available regarding the roles of mechanical force in immune/inflammatory regulation of periodontal tissue. For example, it has been reported that lack of occlusal stimuli in rat models induces atrophic changes of the PDL with upregulation of IL-1β expression, while downregulation of IL-1β expression is observed after restoration of occlusion (44), suggesting that cyclic mechanical stimulation can regulate the expression of IL-1β in periodontal tissue. In the present study, we demonstrated for the first time that cyclic stretch induced PDL cells to secrete exosomes, which exhibited anti-inflammatory properties in macrophages. We previously reported that the exposure of LPS-primed macrophages to cyclic stretch inhibited IL-1β secretion by attenuating the AMP kinase pathway (45). Therefore, the NLRP3 inflammasomes of macrophages may be tightly regulated by multiple feedback systems that maintain immune/inflammatory homeostasis in periodontal tissue.

A previous study reported that exosomes are released in other physiological mechanical environments. In the vasculature, shear stress stimulates human umbilical vein endothelial cells to secrete exosomes, which are, in turn, transferred to smooth muscle cells to induce the atheroprotective smooth muscle cell phenotype (10). In the heart, mechanical stretch induces cardiomyocytes to release exosomes, which are taken up by cardiac fibroblasts and function to suppress myocardial fibrosis (9), suggesting that physiological mechanical force elaborately regulates cell-cell communication through the release of exosomes that contribute to physiological tissue remodeling as well as inflammatory/immune homeostasis in respective tissues/organs. Cyclic stretch-induced exosome secretion was not observed in other dental mesenchymal cells, such as dental pulp cells and GF. Since dental pulp cells and GF are not physiologically exposed to cyclic stretch force, the cyclic stretch-mediated exosome secretion system may be selectively equipped with PDL cells to maintain periodontal homeostasis. On the other hand, exosomes have been detected in the supernatants of normal cultures of highly differentiated dental pulp cells (46) and stem cells from human exfoliated deciduous teeth (47, 48), both of which are derived from the dental papilla of the tooth germ, similar to the dental pulp cells used in the present study. The different statuses of differentiation stages and surrounding microenvironments may be reasons for this discrepancy.

Since exosomes are enriched with nucleic acids, proteins, and lipids, the transfer of these bioactive materials to target cells through internalization by way of fusion and/or endocytosis (49) may be one of the mechanisms responsible for the effects observed. Since a growing number of microRNAs have been implicated in the regulation of the NF-κB signaling pathway (50, 51), the genetic information contained in PDL cell-derived exosomes needs to be clarified in further studies in terms of the expression of microRNAs associated with the regulation of NF-κB signaling. On the other hand, tumor-derived exosomes have been shown to regulate T-cell functions by mechanisms that are dependent on cell surface signaling and do not require exosome internalization by target cells (52). Thus, exosomes may interact with target cells through several different mechanisms, such as fusion with the plasma membrane, micropinocytosis, phagocytosis, clathrin-mediated, caveolin-dependent, lipid raft-dependent endocytosis, receptor-mediated endocytosis, and receptor/ligand interactions (53). In this study, we demonstrated that treatment with cytochalasin D, an inhibitor of actin polymerization that suppresses exosome uptake (54), restored the exosome-mediated inhibition of NF-κB activation, suggesting that an endocytic process may be required for the inhibitory effects of exosomes. However, exosomes from PDL cells may transmit co-stimulatory signals into target cells through receptor/ligand interactions in parallel with the endocytic process for exosome-mediated NF-κB inhibition. Since exosomes may utilize several different mechanisms of uptake in the same cell and at different times, further research needs to be conducted.

Exosomes have emerged as a potential cell-free therapeutic tool in a wide range of diseases (55). Previous studies reported that exosomes from mesenchymal stem cells (MSC) ameliorate systemic inflammation in several animal models, such as acute liver failure and the neuroinflammation associated with post-traumatic brain injury (21–23). Indeed, exosomes derived from MSC have been implicated in many aspects of the cell-based MSC therapeutic potencies, and exosomes have potential as a therapeutic strategy due to their many advantages, such as greater stability, lower possibility of immune rejection, no risk of transformation into inappropriate cell types, and no risk of persistence as permanent grafts upon the cessation of therapy (56). A recent study reported that an intravenous injection of exosomes purified from the conditioned medium of a static culture of human PDL cells suppressed experimental autoimmune encephalomyelitis (EAE), a mouse model of multiple sclerosis, by reducing pro-inflammatory cytokines in the spinal cord (57). Although the underlying molecular mechanisms were not analyzed in that study, the mechanism elucidated in the present study may partly contribute to reversing the progression of EAE. However, the molecular composition of exosomes varies not only with the cell type and origin, but also with the cell activation/differentiation status, even in the same parental cells (58). It also differs depending on the cell site of origin, as observed for epithelial cells; epithelial exosomes have a different composition if they are released from the apical or basolateral surfaces (59). Therefore, exosomes purified from a normal static culture of PDL cells may exert different effects on their target cells from those purified from cyclic stretch-exposed PDL cells. We also demonstrated that cyclic stretch-PDL cells have potential as a potent inducer of anti-inflammatory exosomes, the yield of which was ~6.62 ± 0.46 μg of exosomes/10^6^ cells, whereas a static culture of PDL cells exhibited only the marginal release of exosomes, which was approximately 30-fold less than that of the cyclic stretch culture. In the majority of studies in which exosomes purified from cell-conditioned medium are used, there has been no description of the yield of exosomes. Although there are fewer studies on MSC, <1.0 μg of exosomes was reported to be recovered from 10^6^ cells of a static culture of mouse/rat bone marrow-derived MSC (60–62), suggesting that cyclic stretch-exposed PDL cells very efficiently release exosomes. Since exosomes have emerged as a potential cell-free therapeutic tool for a wide ranges of diseases (55), a strategy to increase the yield of desired exosomes for use in therapeutics is important, and, in order to achieve this, future studies are needed to clarify the molecular mechanisms by which mechanical signals efficiently induce exosome release.

In conclusion, the present results not only provide a potentially novel mechanism for physiological mechanical force contributing to inflammatory/immune homeostasis through the release of exosomes in periodontal tissue, but also suggest the possible utility of PDL cell exosomes as therapeutic tools for a wide ranges of inflammatory diseases.

## Ethics Statement

Experimental procedures were approved by the Ethical Review Board of Tohoku University Graduate School of Dentistry approval number 26–27.

## Author Contributions

ZW and EN: study conception and design. ZW, KM, YS, and SS: acquisition of data. ZW, KM, YS, SS, HT, MiS, MaS, SY, and EN: analysis and interpretation of data. ZW and EN writing the manuscript. EN: final approval of the article.

### Conflict of Interest Statement

The authors declare that the research was conducted in the absence of any commercial or financial relationships that could be construed as a potential conflict of interest.
