# Supplementary figures and images for: Cyclic Stretch Force Induces Periodontal Ligament Cells to Secrete Exosomes That Suppress IL-1β Production Through the Inhibition of the NF-κB Signaling Pathway in Macrophages
Source: Front Immunol. 2019 Jun 20;10:1310. doi: 10.3389/fimmu.2019.01310 (PMC6595474; doi:10.3389/fimmu.2019.01310)

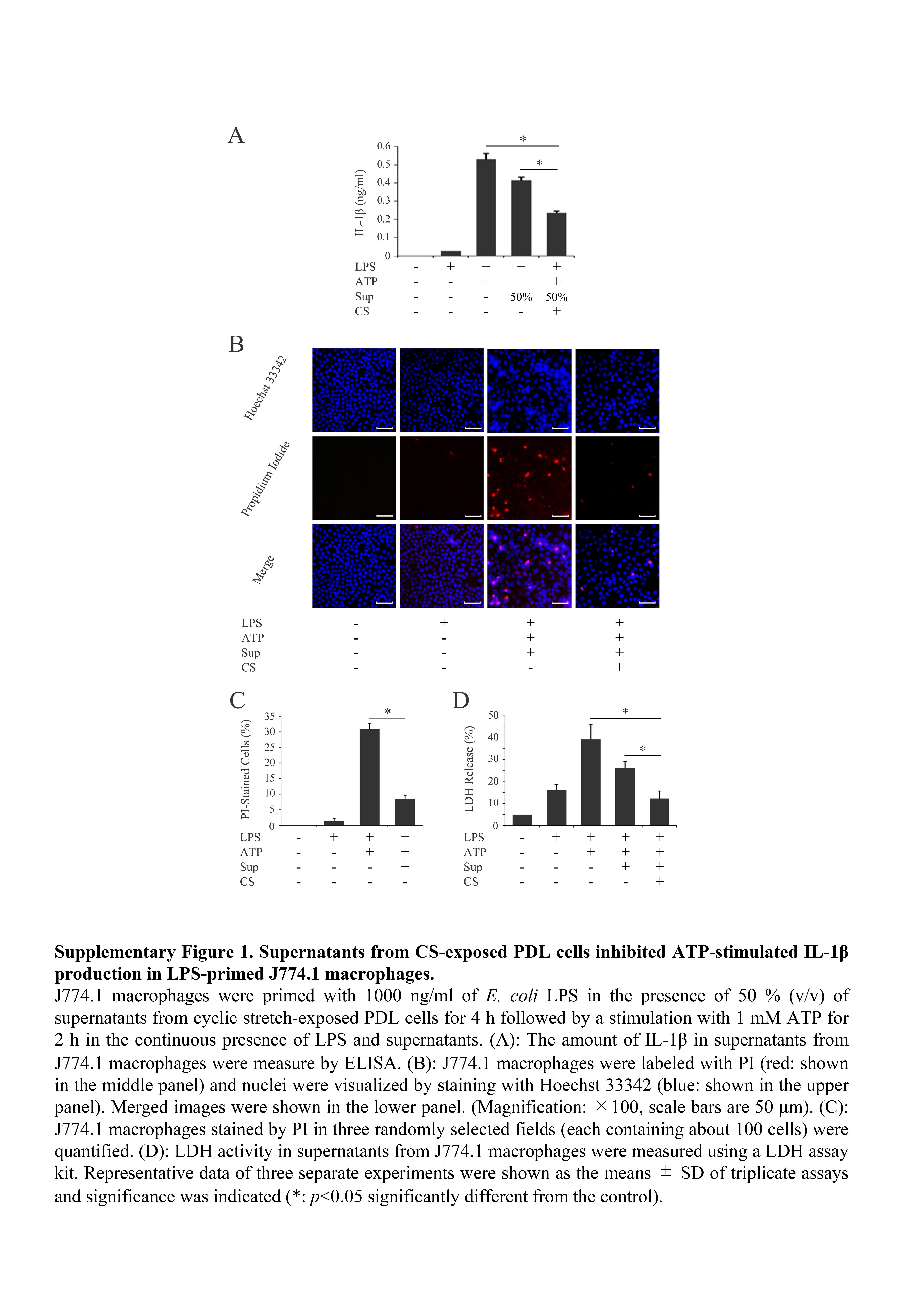

Supplement: Supplementary file 1 [file Image_1.JPEG]

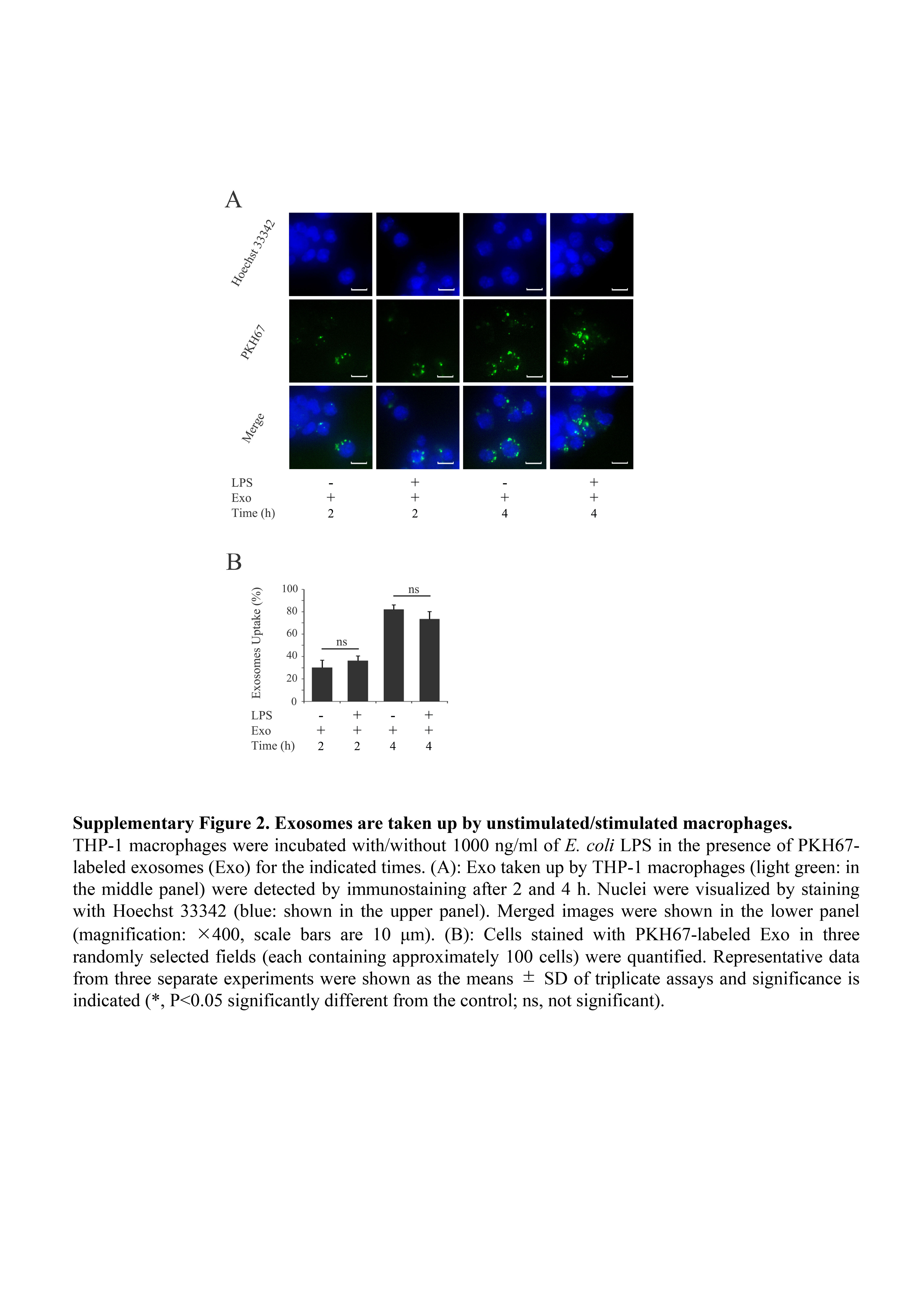

Supplement: Supplementary file 2 [file Image_2.JPEG]

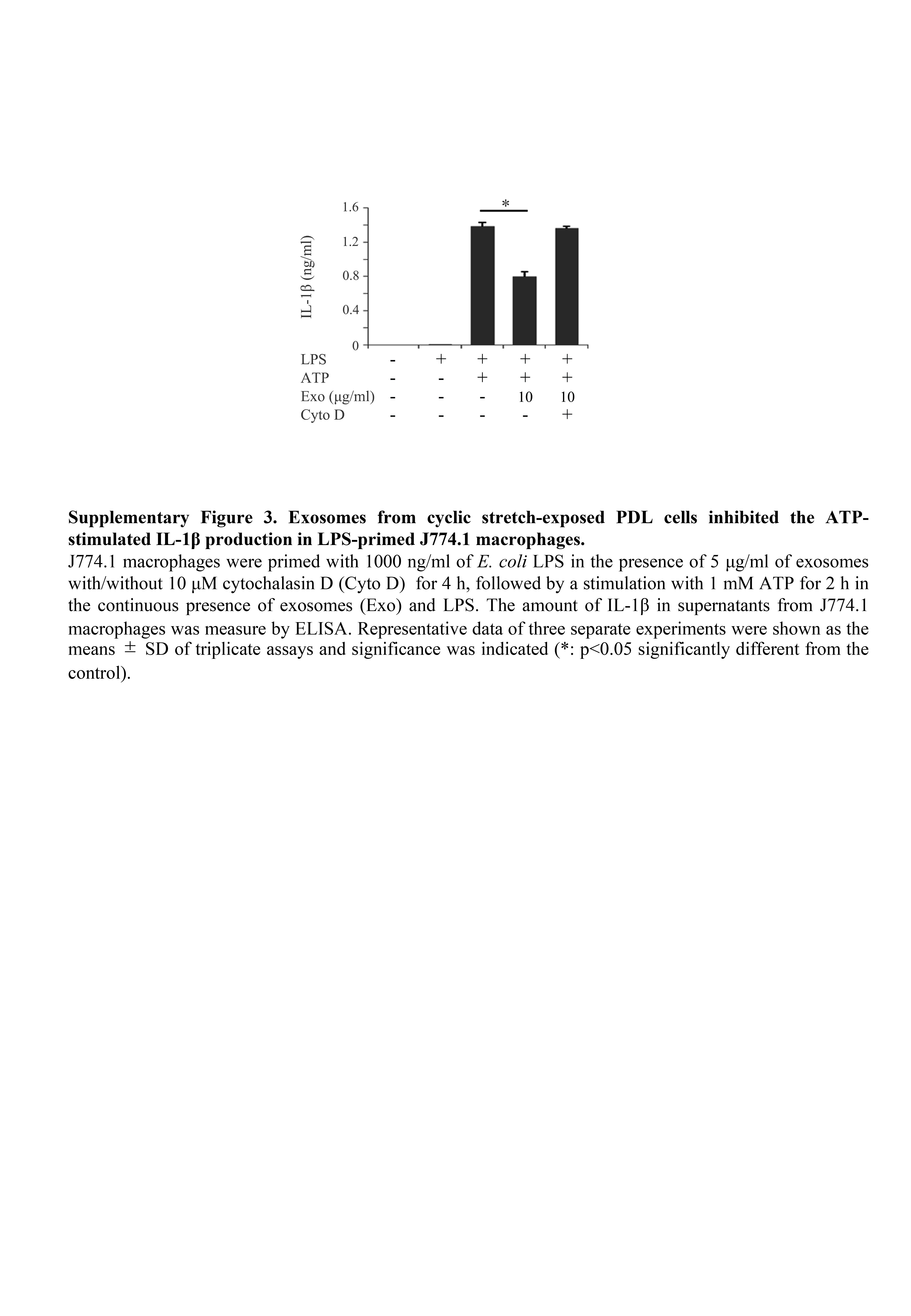

Supplement: Supplementary file 3 [file Image_3.JPEG]

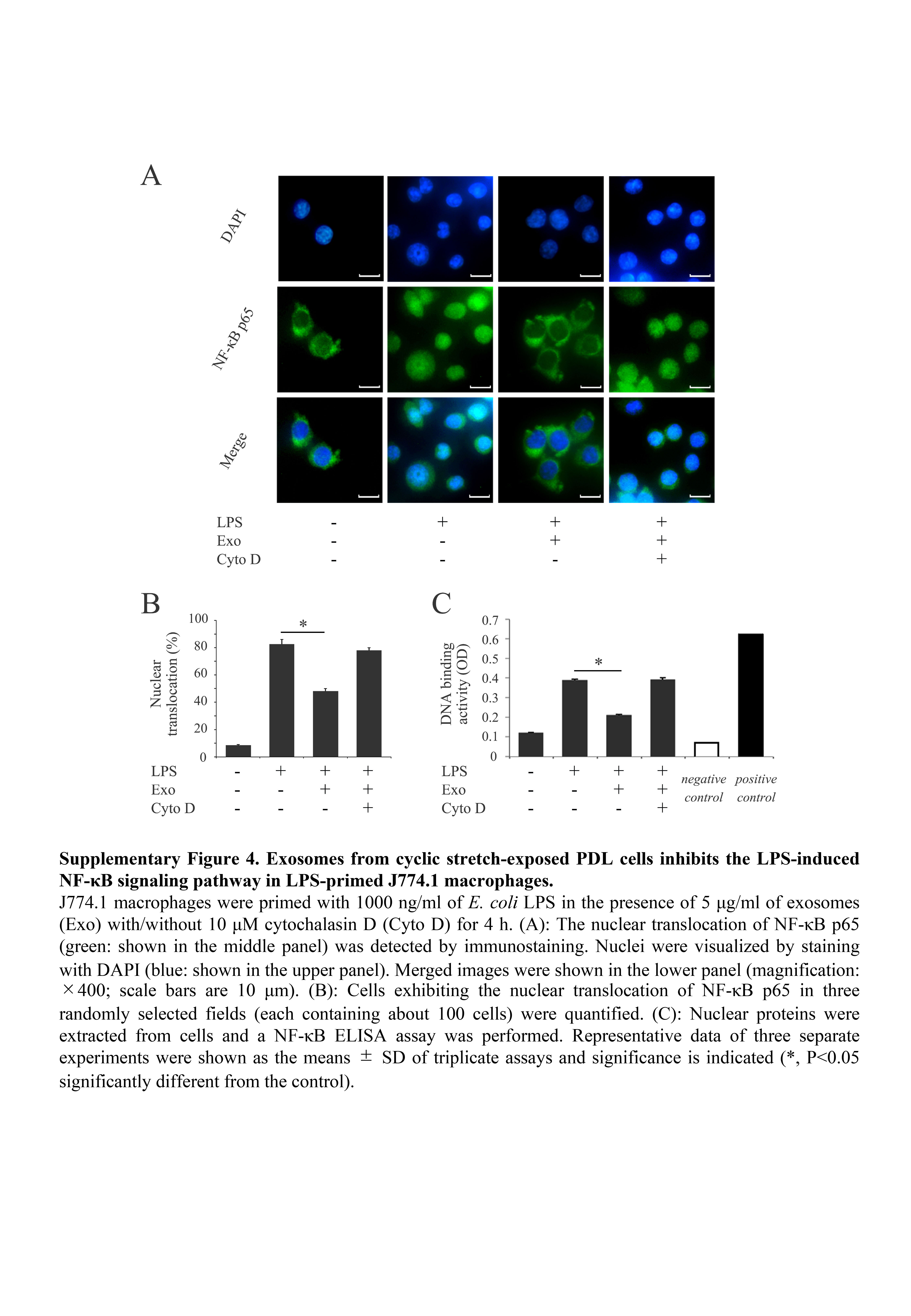

Supplement: Supplementary file 4 [file Image_4.JPEG]
